# Biochar and urease inhibitor mitigate NH_3_ and N_2_O emissions and improve wheat yield in a urea fertilized alkaline soil

**DOI:** 10.1038/s41598-021-96771-0

**Published:** 2021-08-31

**Authors:** Khadim Dawar, Shah Fahad, M. M. R. Jahangir, Iqbal Munir, Syed Sartaj Alam, Shah Alam Khan, Ishaq Ahmad Mian, Rahul Datta, Shah Saud, Jan Banout, Muhammad Adnan, Muhammad Nauman Ahmad, Aamir Khan, Raf Dewil, Muhammad Habib-ur-Rahman, Mohammad Javed Ansari, Subhan Danish

**Affiliations:** 1 Department of Soil and Environmental Science (SES), The University of Agriculture, Peshawar, KPK Pakistan; 2 Department of Agronomy, The University of Haripur, Haripur, Khyber Pakhtunkhwa 22620 Pakistan; 3 Hainan Key Laboratory for Sustainable Utilization of Tropical Bioresource, College of Tropical Crops, Hainan University, Haikou, 570228 Hainan China; 4 Department of Soil Science, Bangladesh Agricultural University, Mymensingh, 2202 Bangladesh; 5 Institute of Biotechnology and Genetic Engineering, The University of Agriculture, Peshawar, Pakistan; 6 Department of Plant Pathology, The University of Agriculture, Peshawar, Pakistan; 7 Department of Plant Protection, The University of Agriculture, Peshawar, Pakistan; 8 Department of Geology and Pedology, Faculty of Forestry and Wood Technology, Mendel University in Brno, Zemedelska1, 61300 Brno, Czech Republic; 9 Department of Horticulture, Northeast Agricultural University, Harbin, China; 10 Faculty of Tropical AgriSciences, Czech University of Life Sciences Prague, Prague, Czech Republic; 11 Department of Agriculture, University of Swabi, Swabi, Khyber Pakhtunkhwa Pakistan; 12 Department of Agricultural Chemistry, The University of Agriculture, Peshawar, Pakistan; 13 Process and Environmental Technology Lab, Department of Chemical Engineering, KU Leuven (University of Leuven), Leuven, Belgium; 14 Crop Science, Institute of Crop Science and Resources Conservation (INRES), University of Bonn, Bonn, Germany; 15 Department of Agronomy, MNS University of Agriculture Multan, Multan, Pakistan; 16 Department of Botany, Hindu College Moradabad (Mahatma Jyotiba Phule Rohilkhand University Bareilly), Moradabad, 244001 India; 17 Department of Soil Science, Faculty of Agricultural Sciences & Technology, Bahauddin Zakariya University, Multan, Punjab 60800 Pakistan

**Keywords:** Plant sciences, Environmental sciences, Pollution remediation

## Abstract

In this study, we explored the role of biochar (BC) and/or urease inhibitor (UI) in mitigating ammonia (NH_3_) and nitrous oxide (N_2_O) discharge from urea fertilized wheat cultivated fields in Pakistan (34.01°N, 71.71°E). The experiment included five treatments [control, urea (150 kg N ha^−1^), BC (10 Mg ha^−1^), urea + BC and urea + BC + UI (1 L ton^−1^)], which were all repeated four times and were carried out in a randomized complete block design. Urea supplementation along with BC and BC + UI reduced soil NH_3_ emissions by 27% and 69%, respectively, compared to sole urea application. Nitrous oxide emissions from urea fertilized plots were also reduced by 24% and 53% applying BC and BC + UI, respectively, compared to urea alone. Application of BC with urea improved the grain yield, shoot biomass, and total N uptake of wheat by 13%, 24%, and 12%, respectively, compared to urea alone. Moreover, UI further promoted biomass and grain yield, and N assimilation in wheat by 38%, 22% and 27%, respectively, over sole urea application. In conclusion, application of BC and/or UI can mitigate NH_3_ and N_2_O emissions from urea fertilized soil, improve N use efficiency (NUE) and overall crop productivity.

## Introduction

In terrestrial ecosystems, nitrogen (N) deficiency is the main growth limiting factor, thereby adversely affecting crop productivity^1–4^. Therefore, an exogenous supply of N along with phosphorus as fertilizers is key farming practice for improving crop yield^3^. Conferring to^5^, a huge quantity of chemically synthetized N compounds will be required to fulfill the food requirements of 9 billion people by 2050. However, the higher land application of N compounds as chemical fertilizers has a negative influence on the environment through the release of N containing gasses such as NH_3_ and N_2_O, and its losses to the ground and surface water by nitrate leaching and runoff^6–8^. At current, the N use efficiency of synthetic N fertilizers is too low, only amounting approximately 50%^9,10^, which adds to the adverse environmental, agronomic and economic impact^11,12^.

Ammonia volatilization is a major source for N losses and is considered to be the main cause of low N use efficiency^9,13^. Ammonia is a major alkaline atmospheric pollutant that plays an important role in the formation of aerosols^14,15^, which badly affect human health^16^, reduce visibility^17,18^, alter Earth’s radiative balance, and contribute to a global redistribution of N through atmospheric deposition^19,20^. Although ammonia is not considered a potential greenhouse gas (GHS), its emission and re-deposition can adversely affect the environment^21^, and it may act as a secondary cause of N_2_O emission in soil^22,23^.

Nitrous oxide is a potential (150 years lifetime) GHS, which is 298 times more efficient than CO_2_ for its heat-trapping capacity with a 7% contribution to the total GHS emission and 0.26% annual growth rate^24^. Agricultural soils annually emit 4.1 Tg N to the global atmospheric N_2_O emission of 14 Tg N^24,25^. In agricultural soils, N_2_O is emitted mainly by biological and chemical processes such as nitrification and denitrification^26–28^. Additionally, N_2_O emissions may also happen via nitrification–denitrification through autotrophic NH_3_-oxidizing bacteria, where ammonia is oxidized to NO_2_^−^, followed by its reduction into NO, N_2_O and N_2_^26,29,30^. It can also be produced during a hybrid reaction by co-denitrification where two N atoms are released one each from organic N by mineralization and NO_2_^−^ by denitrification^31^. In soil containing high organic matter, it can be released by heterotopic reduction of nitrite by the oxidation of organic N^32,33^.

Various studies have been aiming to develop sustainable and eco-friendly management practices for reducing N losses, improving NUE and crop yield^34,35^. Such practices include application of nitrification inhibitors, urease inhibitor (UI), elemental S and polymers^36^, removal of crop residues from the land in various ratios^37,38^ and application of biochar^39,40^. Biochar, a carbon-rich pyrolitic product of organic waste, has received ample attention for its environmental benefits^41^. Utilization of biochar as a soil amendment/conditioner not only provides a way to recycle environmental waste but also optimizes soil health, crop yield and stimulates soil C sequestration^42–50^. Studies have demonstrated that biochar may be used as a compound fertilizer in conjunction with mineral sources^51,52^, as a slow-release fertilizer^53–55^, which could enhance fertilizer^56^ and may increase N use efficiency under different soil conditions^51,52^. Biochar may have the capacity to sorb NH_4_^+^ or NH_3_ gas released during composting, thereby lowering NH_3_ emission. Biochar may also adsorb organic N compounds, thus decreasing their mineralisation^57^ and consequently NH_3_ emission. This gradual availability of N may be partially explained by lower atmospheric N_2_O emissions from N fertilizer applied in the presence of biochar^58,59^.

The application of UI [N-(n-butyl)] and thiophosphoric triamide (nBTPT or NBPT for short) have been reported to reduce urease activity in soil and slow down NH_4_^+^ release from urea, thus reducing NH_3_ emission^34,35,60^. Urease inhibitor significantly decreases urea hydrolysis, reduced NH_4_^+^ concentrations and, thereby limited NO_3_^−^ supply and nitrification rate thus reducing N_2_O emission and improves crop yield^61–64^.

So far, many experiments have focused the effect of biochar and urease inhibitor sole application on gaseous N emissions, N-transformation processes and crop yield in variable agro-climatic circumstances. However, limited work is documented regarding the influence of urea treated with urease inhibitor and biochar or alone on N_2_O and NH_3_ emission from arable agricultural system under hot climate of Pakistan. Therefore, main objectives of current study were to explore the effect of urea together with urease inhibitor and biochar or alone on crop productivity, N_2_O and NH_3_ emission and N efficiency.

## Results

### Soil mineral N dynamics

**Figure 1:**
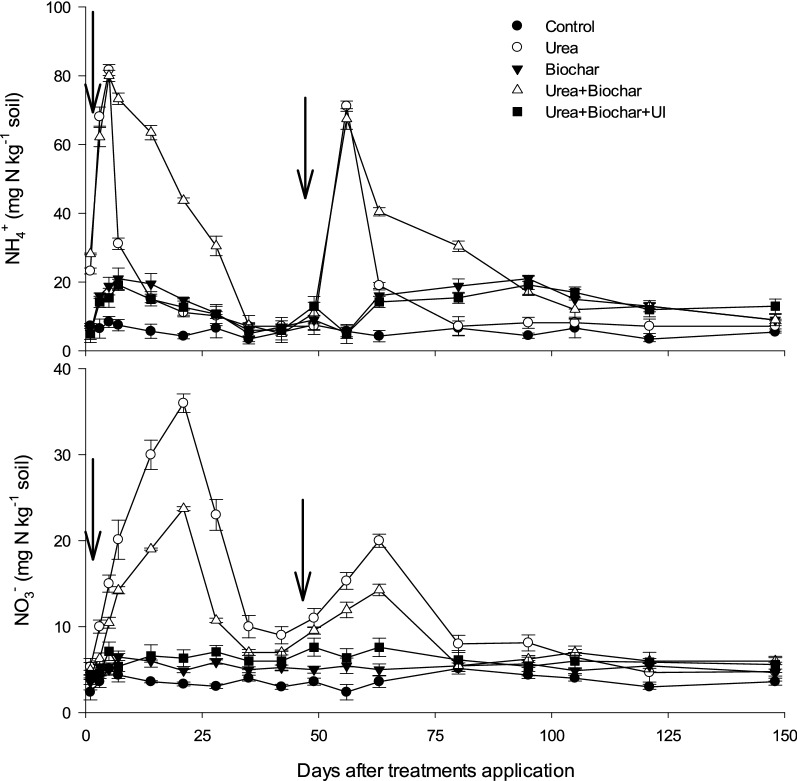
Soil NH_4_^+^ and NO_3_^−^ contents as affected by nitrogen application with and without biochar and/or urease inhibitor. Bars on means represent SE for n = 4. The arrows show time of N application.

The NH_4_^+^-N concentration observed in urea treated plots on day 1 was significantly (p < 0.05) higher (5–25 mg N kg^−1^ soil) compared to the control (Fig. 1). In urea treatment, the NH_4_^+^-N concentration increased because of the applied urea's fast hydrolysis during the first 3 days after first fertilization. Thereafter, a sharp decrease was observed (Fig. 1). The plots treated with biochar + urea showed maximum soil NH_4_^+^-N concentration 28 days after fertilization, which was significantly higher than the plots treated with sole urea (Fig. 1). Furthermore, the application of UI considerably decreased the average soil NO_3_^−^-N content. After the first fertilization, soil NO_3_^–^-N content slightly increased, and most mineral N was observed as NH_4_^+^-N. Soil NO_3_^–^-N concentration peaked just after the second urea application in the urea treatment and then displayed a rapidly decreasing trend within 14 days in after fertilizer application (Fig. 1). Additionally, biochar supplementation significantly decreased NO_3_^–^-N concentration in the soil.

### Ammonia volatilization

**Figure 2:**
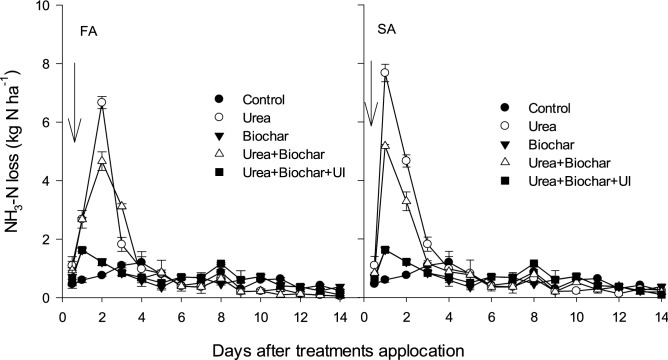
Soil NH_3_ Fluxes as influenced by nitrogen application with and without biochar and/or urease inhibitor. Bars on means represent SE for n = 4. The arrows show time of N application. Where, FA (First dose application) and SA (Second dose application).

The results concerning NH_3_ emission are presented in Fig. 2 and Table 1. A maximum NH_3_ discharge was detected between days 1 and 3 after every application. The application of BC and UI with urea significantly affected daily and total cumulative NH_3_ discharge during the initial 10 to 12 days. Total NH_3_ losses as kg N ha^−1^ amounted 14.4 with BC and 6.2 with BC + UI compared to sole urea (19.7), representing 27% and 69% reduction by BC and BC + UI, respectively, relative to sole urea application.

**Table 1:** Nitrogen losses as ammonia volatilization as affected by nitrogen application with and without biochar and/or urease inhibitor.

Treatments	NH_3_-N losses (kg ha^−1^)	N lost as NH_3_ (% of the applied N)	% Changes in NH_3_ relative to urea
Control	0.7 ± 0.12^a^	–	–
Urea	19.7 ± 0.12^e^	12.7	–
Biochar	4.5 ± 0.12^b^	2.5	–
Urea + biochar	14.4 ± 0.12^d^	9.1	− 27
Urea + biochar + UI	6.2 ± 0.12^c^	3.7	− 69

Means followed by same letter(s) within columns are statistically non-significant (p < 0.05).

### Nitrous oxide emissions

**Figure 3:**
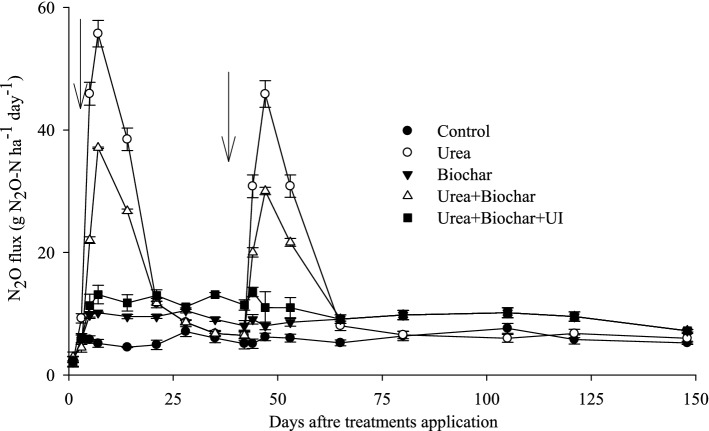
Soil N_2_O flux in response to nitrogen application with and without biochar and/or urease inhibitor. Bars on means represent SE for n = 4. The arrows show time of N application.

The application of urea, BC, and UI significantly affected the daily and cumulative N_2_O emissions (Fig. 3 and Table 2). The highest and lowest cumulative emissions of N_2_O were measured for urea and urea + BC + UI treatments, respectively (1.7 and 0.8 kg N_2_O-N ha^−1^) (Table 2). Biochar + urea significantly reduced N_2_O emissions by 24% compared to urea alone. Urea applied in combination with BC and UI reduced N_2_O emission by 53% over urea alone treatment.

**Table 2:** Nitrogen loses as nitrous oxide in response to urea supplementation with and without biochar and/or urease inhibitor.

Treatments	Total N_2_O losses (kg ha^−1^)	N lost as N_2_O (% of the applied N)	% Changes in N_2_O relative to urea
Control	0.4 ± 0.12^a^	–	–
Urea	1.7 ± 0.34^e^	1.4	–
Biochar	0.6 ± 0.15^b^		–
Urea + biochar	1.3 ± 0.22^d^	1.0	− 24
Urea + biochar + UI	0.8 ± 0.17^c^	0.40	− 53

Means followed by same letter(s) within columns are statistically non-significant (p < 0.05).

### N-use efficiency, yield and N uptake by wheat

Wheat N uptake, yield and NUE were significantly improved due to biochar and/or UI application (Table 3). Wheat biomass (24%) and grain yields (13%) were improved by BC over sole urea. In contrast, urea's combined application with BC and UI increased wheat biomass and grain yields by 38% and 22% over the sole urea treatment. The highest total N uptake, above ground wheat biomass, contained 101 and 114 kg N ha^−1^ from urea + BC and urea + BC + UI treated plots, respectively (Table 3). The BC and UI application increased total N uptake by 12% and 27%, respectively, over sole urea (Table 3). Similarly, the highest N uptake and NUE were observed in plots treated with urea + BC + UI, followed by urea + BC (Table 3). The NUE observed for sole urea, urea + BC and urea + BC + UI were 27%, 35%, and 43%, respectively (Table 3).

**Table 3:** Wheat biomass and grain yields (kg ha^−1^), N uptake (kg ha^−1^) and NUE (%) as affected by urea fertilization with and without biochar and/or urease inhibitor.

Treatments	Biomass yield (kg ha^−1^)	% Increase over urea	Grain yield (kg ha^−1^)	% Increase over urea	Total N uptake (kg ha^−1^)	% Increase over urea	NUE (%)
Control	5604 ± 62^a^		2683 ± 32^a^		49 ± 11^a^		
Urea	7930 ± 68^c^		3827 ± 47^c^		90 ± 15^c^		27
Biochar	6731 ± 52^b^		3328 ± 38^b^		62 ± 0.09^b^		9
Urea + biochar	9855 ± 62^d^	24	4327 ± 46^d^	13	101 ± 17^d^	12	35
Urea + biochar + UI	10,978 ± 58^e^	38	4651 ± 54^e^	22	114 ± 21^e^	27	43

Means followed by same letter(s) within columns are statistically non-significant (p < 0.05).

## Discussion

### Ammonia volatilization as affected by biochar and urease inhibitor

A 27% decrease was observed in NH_3_ volatilization with BC compared to sole urea (Table 1), which can be attributed to its highly porous structure, surface area and high sorption capacities^60,61^. Furthermore, BC may absorb NH_4_^+^-N^62^ due to the presence of acidic functional groups^63^, hence decreasing the NH_3_ volatilization^64^. Biochar application with N fertilizers could avoid soil alkalization^65,66^ and decrease organic N mineralization through the adsorption of organic N compounds^32,57^.

On day 1 after application, a rapid increase in NH_4_^+^-N content was observed with sole application of urea (Fig. 1), which could be ascribed to the fast hydrolysis of urea. As a result, more NH_4_^+^-N and OH^−^ ions are produced and allowing significant NH_3_ losses (Fig. 1). Urease inhibitor reduce the effect of pH increasing by delaying urea hydrolysis, as demonstrated by lower NH_4_^+^-N (Fig. 1) which could significantly reduce NH_3_ emissions. Slower urea hydrolysis due to UI application could also be associated with increasing pH around urea particles, which prevents hydrolytic action on urea via the enzyme urease^10,35,67^. It has also been reported that UI slows down the microbial decomposition of ammonia^68–71^. Additionally, slower urea hydrolysis due to application of UI could also provide more time for rainfall or irrigation to move the applied urea from the soil surface to the sub-soil layers vertically as well as and laterally consequently protects the applied N from volatilization^72,73^.

### N_2_O emission as affected by biochar and urease inhibitor

Nitrous oxide emissions are directly related to the amount of mineral N available in the soil. A two-way ANOVA indicated that seasonal N_2_O emissions during the wheat growing season were significantly affected by biochar application (Fig. 2), in agreement with previous results Zhang et al.^33^ and Schirrmann et al.^74^. Our results confirm that the application of biochar reduced N_2_O emissions, because biochar resulted in lower NO_3_^−^ contents and higher NH_4_^+^ concentrations after N addition (Fig. 2) which affect N availability in the soil, either physically by sorption to surfaces or microbiologically^75^. These results are in agreement with the findings of Lehmann et al.^76^ and Kammann et al.^77^ who reported that after application of N fertilizer, the adsorption of soil NH_4_^+^ by biochar on its surface, especially by biochar with a maximum C/N ratio reduce N_2_O emissions, subsequently resulting a decrease in the processes of ammonification and nitrification.

Nitrous oxide is generated by both nitrification and denitrification processes; therefore, it may be closely related to soil NH_4_^+^-N and NO_3_^–^-N concentrations^78^. Under high nitrification activity, when a lot of NO_3_^−^ is produced in urea-treated soils during the first week of treatment application (Fig. 1) the observed N_2_O may actually be related to nitrification which produces N_2_O as a byproduct^10^. Apart from high water content also a high microbial activity can cause O_2_ depletion and therefore anoxic conditions that might promote denitrification. Due to the strong nitrification usually taking place during aerobic conditions, NO_3_^−^ predominates in soils. As a result, denitrification would be prompted by the large amounts of NO_3_^−^ accumulated in soil, especially under high soil moisture conditions. For a given soil, whether nitrification or denitrification contributed more to N_2_O emission may be closely related to soil NH_4_^+^-N and NO_3_^−^-N concentrations when the initial physicochemical factors of the tested soil are almost identical^79^.

The results showed lower NH_4_^+^-N concentrations in plots treated with urea + BC + UI than sole urea treated plots, while NO_3_-N concentrations were higher in urea treated plots than in urea + BC + UI (Fig. 1 and Fig. 3). It is likely that the combination of biochar and UI significantly decreased NO_3_^−^ supply and thus reduced the activity of denitrifiers. Here, it is evident that UI application reduced the activity of ammonia oxidase and nitrification process and played a significant role in converting NH_4_^+^-N to NO_3_^–^-N, which is significantly related to N_2_O discharge from soil^10,36,67^. These findings imply that applying either BC or BC + UI plays a significant role in reducing NH_3_ and N_2_O emissions from urea fertilized soils. Additional investigations are recommended to elucidate the role of BC and BC + UI in reducing NH_3_ and N_2_O emissions at varying soil and climatic conditions.

### Wheat yield and N uptake as affected by biochar and urease inhibitor

Both BC and UI significantly improved total N uptake and yield of wheat (Table 3). Application of BC and/or UI with N fertilizers can reduce N losses as GHS and may prevent NO_3_^−^ leaching, thus enhancing the bioavailability of N^36,48,60^. Dawar et al.^13^ reported that the retention of mineral N in the form of NH_4_^+^, rather than NO_3_^−^, for several days after urea application increasing N uptake and thus improved crop yield. The results also show that BC improved soil WHC, allowing the wheat to retain a proper moisture level between irrigation periods, with a subsequent positive impact on final grain yields^80^. Furthermore, BC + UI could significantly improve soil conditions such as soil organic content, pH, and total N content^81^, improving N availability by reducing NH_3_ and N_2_O from the soil. Urease inhibitor and BC retain NH_4_^+^ in the soil for a longer time and improve its subsequent uptake by wheat, thus may improve crop yield. This not only offers environmental benefits by preventing and NO_3_^−^ leaching and sinking NH_3_ and N_2_O release^13,36^, but also includes economic benefits using an increase in NUE. The plant uses relatively less energy to absorb NH_4_^+^ than NO_3_^−^ as the transformation of NO_3_^−^ into NH_4_^+^ via amides, amines, and amino acids is energy-consuming. Thus, higher availability of NH_4_^+^ facilitates improved crop yield and nutrition^10^.

## Conclusions

It was observed that BC and UI have the highest potential to reduced NH_3_ and N_2_O emissions in urea fertilized soils. Furthermore, applying BC and/or UI with urea significantly improved wheat biomass and grain yield compared to sole urea. Therefore, the application of BC and/or UI with urea plays a significant role in mitigating NH_3_ and N_2_O emissions from cultivable land and improving crop yield under the hot and semi-arid agro-climatic conditions of Pakistan.

## Materials and methods

### Location

**Figure 4:**
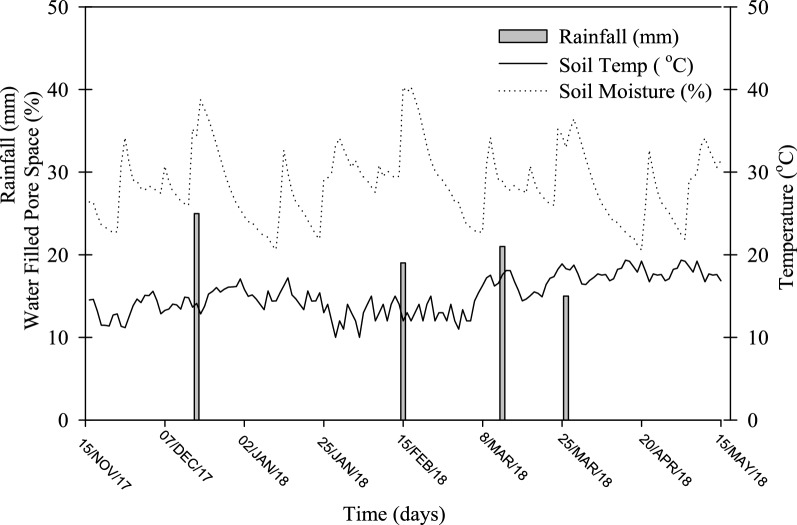
Soil (0–10 cm) temperature, moisture and precipitation throughout the growing season.

This experiment was executed at the National Institute of Food and Agriculture (NIFA), Peshawar (34.01°N, 71.71°E), Pakistan. The study area is semi-arid to arid and humid in the north to dry in the southern parts with a mean yearly precipitation of 384 mm and temperature of 22.7 °C (Fig. 4). The field had been conventionally cultivated with maize-wheat cropping system for approximately 10 years. The main soil (0–15 cm) properties of the composite sample are provided in Table 4.

**Table 4:** Characterization of experimental field.

Property	Quantity
Texture	Silt loam
Electrical conductivity (EC)	0.26 d Sm^−1^
pH	7.80
Soil Bulk density (BD)	1.27 g cm^−3^
Organic matter (OM)	0.88%
AB-DTPA extractable P	2.42 mg kg^−1^
Total nitrogen	0.089%

### Experiments

The field experiment including five treatments [control; urea (150 kg N ha^−1^); BC (10 t ha^−1^); urea + BC and urea + BC + UI] was designed following a randomized complete block design (RCBD) with 4 replications. The ‘Pirsabak-2013’ variety of wheat was sown in 5 m × 3 m plots containing 10 rows with row-to-row distance was kept 30 cm apart at a seed ratio of 120 kg ha^−1^ on 15th Nov, 2017. The conventional tillage was implemented using a mouldboard plow (40–50 cm deep) followed by disking (20–25 cm deep) and land leveller operations for seed bed preparation. Before seeding, 90 kg P ha^−1^ and 60 kg K_2_O ha^−1^ as single super phosphate and sulphate of potassium, respectively, were applied as a basal dose. The biochar used was produced from eucalyptus wastes (branches) pyrolyzed at 350 °C and sieved through a 5 mm sieve. Nitrogen was supplemented in the form of urea as a top dressing in two splits, half each a sowing and tillering. Urease inhibitor was supplemented as 0.1% solution. Granular urea with UI containing 25% NBPT was added at 150 kg N ha^–1^ as a surface application followed by surface incorporation and irrigation (10 mm) to ensure its proper distribution in the root zone.

### N_2_O measurement

The N_2_O discharge from soil was measured from November, 2017 to May, 2018 using opaque manual spherical static chambers, following the procedure described in Sanz-Cobena et al.^34^. For sample collection, the cylindrical (radius 12.5 and height 20 cm) chambers were inserted in the soil at 15 cm depth 24 h before sampling. The chambers were properly sealed, and samples were taken after 0, 30 and 60 min once a week in the morning between 7:00 and 10:00 by a butyl septum installed on the upper part of the chamber. The samples were injected in 20 ml glass vials (Agilent Technologies, USA) via syringes (50 ml) equipped with inner three-way stopcocks (0.7-mm). The air and soil temperatures inside the chambers were recorded during gas sampling via a portable digital thermometer. Gas samples were analyzed on gas chromatographs (Varian Aerograph Series 2800 in NZ; Perkin Elmer Auto system XL B5902) equipped with 63Ni electron capture detectors (Pye Unicam) and two manual switching valves (Valco Instruments Co., Inc.)

The mean variation in gas concentration was calculated through linear regression while the ideal gas law was applied for quantification of gas-fluxes as follows:

1 $${\text{F}} = \uprho \times \left( {{\text{P}} / {76}0} \right) \times \left( {{\text{V}} / {\text{A}}} \right) \times \left( {\Delta {\text{C}} / \Delta {\text{t}}} \right) \times \left[ {{273} / \left( {{273} + {\text{T}}} \right)} \right]$$

where F, P, V, A, Δc/Δt and T represent N_2_O flux (µg m^−2^ h^−1^), density (mg m^−3^), chamber size (m^3^), base size (m^2^) of the chamber, mean rate of variation in gas concentration per unit time (mg kg^−1^ h^−1^) and chamber inner temperature (°C), respectively. The acceptable range of R^2^ for N_2_O fluxes was taken as 0.80 for the static chamber, except where the maximum change in concentration was lower than the gas-specific GC detection limit (< 10 ppb for N_2_O), where no filtering criterion was adopted (Järveoja et al.^82^). Using these criteria, 10% of fluxes (N_2_O) were subtracted from succeeding data analysis.

The following formula was used for the determination of cumulative N_2_O emissions (Ec, kg N_2_O-N ha^−1^):

$$\sum_{i=1}^{n}\left({F}_{i}+{F}_{i+1}\right) /2\times ({t}_{i+1}-{t}_{i})\times 24$$

where Fi and Fi + 1 is the ith and (i + 1)th measured value, respectively, of N_2_O flux (µg N_2_O-N m^−2^ h^−1^ ); ti and ti + 1 is the day when the ith and (i + 1)th measurement of N_2_O flux is taken, respectively (d); and n is the total number of the measurements.

### Ammonia measurement and analysis

Ammonia volatilization was quantified by a 5 L (plastic bottle) semi-static open chamber, according to Araujo et al.^83^. In each plot, a single chamber was installed 5 mm above the ground surface and was randomized daily as advised by Jantalia et al.^84^. A foam strip pre-soaked in 1 molar H_2_SO_4_ and 4% (V/V) glycerol was kept moist throughout sampling duration by immersing one end into a polypropylene jar containing 15 mL 1 molar H_2_SO_4_ solution suspended inside the chamber. In each plastic bottle, the solution and foam strip were initially replaced by fresh solution after 12 h and then every 24 h and 14 days and processed for NH_4_^+^-N concentration via the steam distillation method^85^.

The following equation was applied for the quantification of NH_3_ fluxes (kg N ha^−1^ d^−1^):

$$\mathrm{F}=\frac{2 \times \mathrm{C}\times \mathrm{V}\times 14\times {10}^{-2}}{\uppi \times {\mathrm{r}}^{2}}\times \frac{24 }{\mathrm{t}}$$

where; C, V, t and r represent the molar concentration of H_2_SO_4_, amount of H_2_SO_4_ consumed during titration (ml), sampling time (hours) and chamber radius (m). The cumulative NH_3_ flux was determined by adding the NH_3_ fluxes for all sampling days for their respective treatment plots.

This method showed 57% NH_3_ recovery by calibration with ^15^N isotope equilibrium technique (Araújo et al.^83^). Therefore, for the accurate estimation of cumulative NH_3_ emissions and flows, a correction factor of 1.74 was applied (Araújo et al.^86^). According to Jantalia et al.^84^, this method for quantification of NH_3_ emissions is more suitable than the wind tunnel procedure^35^ for comparison of different treatments.

### Soil and plant analysis

From each plot, five soil samples (0–10 cm) were randomly collected, well mixed, sieved (2 mm) and analyzed for key soil properties (Table 4). To determine mineral N (NO_3_^−^ and NH_4_^+^), extraction was done with 1:5, 2 M KCl for 1 h on a rotary shaker^10^, filtered and analyzed via ultraviolet spectrophotometry (Jenway, 6305 UV/Vise, UK). Soil moisture was measured by oven-drying and was transformed to water-filled pore space (WFPS) using the formula of Li et al.^85^.

Total N in soil and plant samples was determined by the Kjekdhal method of Keeney and Nelson^86^. In this method, 0.2 g of finely ground samples of dry materials were digested with 3 ml of concentrated H_2_SO_4_ in the presence of 1.1 g digestion mixture containing CuSO_4_, K_2_SO_4_ and Se on a heating mantle for about 1 h. The digest was transferred quantitatively to the distillation flask and distilled in the presence of 10 ml of 10 MNaOH solutions. The distillate was collected in 5 ml boric acid mixed indicator solution and then titrated against 0.01 MHCl solution by adding 5 ml boric acid mix Indicator. Using the follow formula total N was calculated.

$$\text{Total} \; \text{Nitrogen} \; \%=\frac{({\text{Sample}}-\text{Blank}) \times 0.005 \times 0.014 \times 100 } {{\text{Weight}} \; {\text{of}} \; {\text{soil}} \times {\text{volume}} \; {\text{made}}}$$

For obtaining the data on grain and biological yield, the central four rows were harvested from each plot at physiological maturity and data were recorded on various agronomical traits (biomass, grain yield and straw yield) and total N uptake in crop. Biomass yield was separated into grain and above ground plant tissue (i.e. shoot and leaves) and record their fresh bulk weight immediately. Five randomly chosen plant tissue sub-samples (ca. 1000 g fresh weight) from each sub-plot; were transferred to sealable plastic bags, and transferred to lab in container with ice to ensure no water losses occur from collected plant tissue. After transporting the plant tissue samples to the lab, fresh weight was immediately recorded. After recording the fresh weight, harvested material was placed in pre-weighed paper bags and dried at 65 °C for 7 days. Dry weights of the plant tissue after 7 days were recorded in order to calculate its moisture content or fraction. The grain yield was adjusted for moisture fraction, prior to obtaining its dry weight, using a moisture tester. For N uptake by above ground plant tissues (i.e. shoot and leaves) and by the grain, the two tissues separately samples were ground to a fine powder (for determination of the total N.

Grains yield was recorded after threshing of wheat plants taken from central four rows of each treatment and then converted into kg ha^−1^ by using the following formula.

$${\text{Grain yield}}\,{\text{(kg}}\,{\text{ha}}^{{ - 1}} {)} = \frac{{\text{Grain yield in four central rows}}}{{{\text{Row}}\, - \,{\text{row }}\,{\text{distance}}\,\times\,{\text{Row}}\,{\text{length}}\,\times\,{\text{No}}{.}\,{\text{of}}\,{\text{rows}}}} \times {{10000}}$$

Biological yield was recorded by harvesting 4 central rows in each plot, dried and weighed and then weight was converted into kg ha^−1^ using the following formula;

$${\text{Biological}}\,{\text{yield}}\,{\text{(kg}}\,{\text{ha}}^{{ - 1}} {)} = \frac{{\text{Biological yield in four central rows}}}{{{\text{Row}}\, - \,{\text{row }}\,{\text{distance}}\, \times\,{\text{Row}}\,{\text{length}}\,\times\,{\text{No}}{.}\,{\text{of}}\,{\text{rows}}}} \times {{10000}}$$

Nitrogen uptake and NUE were then calculated as follows:

$$\text{Total} \; \text{ N} \; \text{uptake}=\frac{{\% \text{N} \; \text{in} \; \text{grains} }\times \text{grain} \; \text{yield } \; (\text{kg } \; {\text{ha}}{^{-1}})}{100}$$

$$\text{NUE} \; (\%) = \frac{\text{Total} \; \text{N}.\text{ uptake }\left(\text{kg} \; \text{ha} {^{{-1}}}\right) \; \text{ in} \; \text{fertilized} \; \text{plot }-\text{ Total} \; \text{N}.\text{ uptake }\left(\text{kg} \; \text{ha} {^{-1}}\right)\; \text{ in} \; \text{control} \; \text{plot }}{\mathrm{N}.\mathrm{ applied } \; \left(\text{kg} \; \text{ha} {^{-1}}\right)}\times 100$$

### Statistical analysis

Standard error (SE) of mean (n = 4) was quantified via descriptive statistics. The replicated data were processed using analysis of variance (ANOVA) followed by least significant difference (LSD) test (P < 0.05) using general linear model (GLM)^87^.

### Plant material collection and use permission

No permission is required for plant material as it was purchased from certified dealer of local area.

### Ethics approval and consent to participate

We all declare that manuscripts reporting studies do not involve any human participants, human data, or human tissue. So, it is not applicable.

### Complies with international, national and/or institutional guidelines

Experimental research and field studies on plants (either cultivated or wild), comply with relevant institutional, national, and international guidelines and legislation.
